# Ezrin Is Required for the Functional Regulation of the Epithelial Sodium Proton Exchanger, NHE3

**DOI:** 10.1371/journal.pone.0055623

**Published:** 2013-02-06

**Authors:** Hisayoshi Hayashi, Atsushi Tamura, Devishree Krishnan, Sachiko Tsukita, Yuichi Suzuki, Hetal S. Kocinsky, Peter S. Aronson, John Orlowski, Sergio Grinstein, R. Todd Alexander

**Affiliations:** 1 Laboratory of Physiology, School of Food and Nutritional Sciences, University of Shizuoka, 52-1 Yada, Shizuoka, Japan; 2 Laboratory of Biological Science, Graduate School of Frontier Biosciences and Graduate School of Medicine, Osaka University, Yamadaoka, Suita, Osaka, Japan; 3 Department of Physiology, University of Alberta, Edmonton, Alberta, Canada; 4 Department of Pediatrics, Yale University School of Medicine, New Haven, Connecticut, United States of America; 5 Department of Internal Medicine, Yale University School of Medicine, New Haven, Connecticut, United States of America; 6 Department of Cellular and Molecular Physiology, Yale University School of Medicine, New Haven, Connecticut, United States of America; 7 Department of Physiology, McGill University, Montreal, Canada; 8 Department of Biochemistry, University of Toronto, Toronto, Canada; 9 Program in Cell Biology, Hospital for Sick Children, Toronto, Ontario, Canada; 10 Department of Pediatrics, University of Alberta, Edmonton, Alberta, Canada; University of Geneva, Switzerland

## Abstract

The sodium hydrogen exchanger isoform 3 (NHE3) mediates absorption of sodium, bicarbonate and water from renal and intestinal lumina. This activity is fundamental to the maintenance of a physiological plasma pH and blood pressure. To perform this function NHE3 must be present in the apical membrane of renal tubular and intestinal epithelia. The molecular determinants of this localization have not been conclusively determined, although linkage to the apical actin cytoskeleton through ezrin has been proposed. We set out to evaluate this hypothesis. Functional studies of NHE3 activity were performed on ezrin knockdown mice (*Vil2^kd/kd^*) and NHE3 activity similar to wild-type animals detected. Interpretation of this finding was difficult as other ERM (ezrin/radixin/moesin) proteins were present. We therefore generated an epithelial cell culture model where ezrin was the only detectable ERM. After knockdown of ezrin expression with siRNA, radixin and moesin expression remained undetectable. Consistent with the animal ultrastructural data, cells lacking ezrin retained an epithelial phenotype but had shortened and thicker microvilli. NHE3 localization was identical to cells transfected with non-targeting siRNA. The attachment of NHE3 to the apical cytoskeleton was unaltered as assessed by fluorescent recovery after photobleaching (FRAP) and the solubility of NHE3 in Triton X-100. Baseline NHE3 activity was unaltered, however, cAMP-dependent inhibition of NHE3 was largely lost even though NHE3 was phosphorylated at serines 552 and 605. Thus, ezrin is not necessary for the apical localization, attachment to the cytoskeleton, baseline activity or cAMP induced phosphrylation of NHE3, but instead is required for cAMP mediated inhibition.

## Introduction

The sodium/proton exchanger (NHE) family of proteins mediates the exchange of extracellular sodium for intracellular protons. The third isoform, NHE3, is one of the eleven known members of this family [1], [2]. Unlike the ubiquitously expressed housekeeping isoform NHE1, NHE3 is localized exclusively to the apical region of renal and intestinal epithelia [3], [4], [5]. This apical localization is prerequisite to its function: the vectorial absorption of sodium, bicarbonate and consequently osmotically driven water from intestinal and renal tubular lumina. These NHE3 driven processes are thought to be essential for the maintenance of both plasma pH and blood pressure. Indeed, genetic ablation of NHE3 in mice leads to acidosis and hypotension [6] consistent with a major role for this transporter in systemic electrolyte, fluid and acid-base homeostasis.

Given its physiological importance, there has been considerable interest in understanding the fundamental mechanisms that govern the trafficking and regulation of NHE3 at the apical membrane of epithelia. Not surprisingly, NHE3 was found to be extensively regulated by a miriad of factors, including numerous hormones and growth factors as well as physical parameters such as extracellular osmolarity, the state of filamentous actin assembly, membrane curvature and surface charge [7], [8], [9], [10], [11], [12], [13]. One second messager that appears to play a major role in regulating NHE3 is cAMP. Agents that increase intracellular cAMP levels inhibit the transporter by affecting not only its catalytic activity but also by enhancing its rate of endocytosis [14], [15], though the precise mechanisms are not fully resolved. Previous findings identified the sodium hydrogen exchanger regulatory factor 1 (NHERF1), which binds to the C-terminus of NHE3, as a molecule critical for cAMP mediated inhibition of NHE3 activity [16]. Ezrin, a member of the ezrin/radixin/moesin (ERM) family of actin interacting proteins, associates with NHERF1 (also known as ezrin binding prtein 50 or EBP50 [17]). Ezrin has also been suggested to participate in this regulation by facilitating the association between the C-terminus of NHE3 and PKA, *i.e.* by acting as a PKA anchoring protein (AKAP) [18]. This NHE3/NHERF/ezrin complex is thought not only to be necessary for the inhibition of NHE3 activity, but has also been postulated to serve as a physical link between NHE3 and the actin cytoskeleton [19], [20]. This latter association retains NHE3 at the apical membrane. The current model of NHE3 apical localization thus predicts that tethering to the apical actin cytoskeleton retains NHE3 at the apical cell surface, this interaction being mediated by NHERF and ezrin [21].

Indeed, ours and other laboratories have demonstrated that an association with the apical actin cytoskeleton is, at least in part, responsible for retention of NHE3 at the apical plasma membrane [22], [23], [24]. The interaction between NHE3 and the apical actin cytoskeleton is known to depend on Rho-GTPase activity [9], [25]. Ezrin, as mentioned above, is known to link transmembrane proteins to the actin cytoskeleton (reviewed in [26]). To perform this function ezrin must be in an active conformation with exposed FERM (4.1/ezrin/radixin/moesin) and actin-binding domains [27], [28]. Rho activation favors an open ezrin conformation [29] and would therefore allow a link between NHE3 and the actin cytoskeleton to be formed. Consistent with the proposed model of NHE3 apical localization, inhibition of Rho-GTPase activity results in the redistribution of NHE3 from the apical membrane into an endomembrane compartment [9], [25]. More recently ezrin has been shown to bind directly to both NHE1 [30] and NHE3 [31]; this latter interaction was also reported to be necessary for NHE3 activity.

Despite the suggestive evidence, the mechanism by which NHE3 is attached to the actin cytoskeleton has yet to be clearly delineated. NHERF and ezrin, the molecules proposed to link NHE3 to the apical actin cytoskeleton, have not definitively been shown to fulfill this role. Evidence for their involvement is largely circumstantial and stems from co-immunoprecipitation [31], [32], [33], *in vitro* binding assays [27], affinity chromatography [34], and functional reconstitution experiments [32], [35] (aimed largely at elucidating the mechanism responsible for PKA dependent inhibition of NHE3, summarized in a review by Weinman *et. al.* [36]). Immunolocalization studies on both the NHERF-1 and the ezrin knockout mouse failed to reveal a relocalization of NHE3 to the basolateral or even endomembrane compartment [37], [38], placing doubt as to the validity of the proposed model.

The lack of an alteration in NHE3 localization in the NHERF-1 knockout animal can be explained by either functional redundancy between NHERF1 and its various paralogs or by direct binding of NHE3 to actin via ezrin. Both possibilities could be evaluated by eliminating the expression of ezrin. Detailed analyses of NHE3 localization and function were not reported for the ezrin knockout animal. We therefore assessed NHE3 expression and activity in the ezrin knockdown mouse (*Vil2* ^kd/kd^) and found only subtle alterations from the ezrin replete control. Interpretation of our results, and other studies performed in a whole animal model is complicated by the possibility of functional redundancy between ezrin and other actin linker molecules. Specifically, radixin and/or moesin may substitute for ezrin, in its absence, providing the necessary linkage to the actin cytoskeleton. We therefore set out to test the proposed model of NHE3-actin cytoskeletal linkage by specifically eliminating ezrin with siRNA in an epithelial cell culture model devoid of the other ERM proteins.

## Materials and Methods

### Materials and Solutions

Nigericin, the acetoxymethyl ester of 2′,7′-*bis*(carboxyethyl)-5(6)-carboxyfluorescein (BCECF), 5-(N-ethyl-N-isopropyl)-amiloride (EIPA), Alexa 488-conjugated phalloidin, Alexa 488-conjugated goat anti-mouse antibody and F(ab) fragment were obtained from Molecular Probes Inc. Forskolin, 3-Isobutyl-1-methylxanthine (IBMX), *O*-phenylenediamine dihydrochloride, dimethylamiloride, benzamil, anti-actin and anti-tubulin antibodies were from Sigma (Sigma-Aldrich Canada Ltd., ON, Canada). Anti-hemagglutinin (HA) mouse antibody and F(ab) fragment were from BabCo. Cy2-, Cy3- and Cy5-conjugated secondary antibodies were from Jackson ImmunoResearch Laboratories, Inc. Polyclonal anti-phosphoserine-552 and -605 NHE3 antibody were generated as described [39]. The polyclonal anti-NHE3 antibody was a kind gift of Dr. Orson Moe. The anti-panERM and the anti-phosphoERM antibodies was obtained from Cell Signaling Technologies. Isotonic Na^+^-rich medium contained (in mM) 70 NaCl, 50 N-methylglucammonium (NMG) Cl, 3 KCl, 1 MgCl_2_, and 20 HEPES-Tris (pH 7.4). Isotonic K^+^-rich media had a similar composition except that NaCl was replaced by KCl. The monoclonal anti-ezrin antibody was from Covance Research Products. The polyclonal anti-E-cadherin antibody was from the Developmental Studies Hybridoma Bank at the University of Iowa and the anti-ZO-1 antibody was from Zymed Laboratories Inc. Anti-EBP50 and E3KARP antibodies were the kind gift of Dr. Anthony Bretscher.

### Ezrin Knockdown Mice

The creation of ezrin-knockdown mice (*Vil2* ^kd/kd^) has been described previously [40]. All animals were bred and kept in a pathogen-free environment and were provided with food and water *ad libitum*. Male homozygous (*Vil2* ^kd/kd^) and wild-type (*Vil2* ^+/+^) littermate mice, 2–32 wk of age, were used for experiments. All animal procedures were carried out in accordance with a protocol approved by the institutional Animal Care and Use Committees of Osaka University and the University of Shizuoka.

### Tissue Preparation

The animals were killed by cervical dislocation and a 3 cm segment of mid –distal colon (50 ∼ 80% length) 4 cm distal to the ileocecal junction was excised and then opened along the longitudinal axis. The mucosal-submucosal preparation, consisting of the mucosa, muscularis mucosa, and submucosal layers was obtained with fine forceps. A 5 cm segment of ileum 3 cm proximal to the ileocecal junction was excised and prepared similarly to the colonic preparation.

### Isc Measurements

The Isc and transmural tissue resistance (Gt) were measured *in vitro* using chambers as previously described [41]. The mucosal-submucosal sheet, from male littermates at 22 weeks of age was mounted vertically between acrylic resin chambers with an internal surface area of 0.196 cm^2^. The temperature of the 10-ml bathing solution in each chamber was maintained at 37°C by a water-jacketed reservoir. The standard bathing solution contained (in mM) 119 NaCl, 21 NaHCO_3_, 2.4 K_2_HPO_4_, 0.6 KH_2_PO_4_, 1.2 MgCl_2_, 1.2 CaCl_2_, and 10 glucose. The solution was gassed with 95% O_2_ and 5% CO_2_ (pH 7.4). The tissue was continuously short-circuited, with compensation for the fluid resistance between the two potential-sensing bridges, by using a voltage-clamping amplifier (CEZ9100; Nihon Kohden, Tokyo, Japan). The transepithelial potential was measured through 1 M KCl-agar bridges connected to a pair of calomel half-cells, the transepithelial current being applied across the tissue through a pair of Ag-AgCl electrodes kept in contact with the mucosal and serosal bathing solutions through a pair of 1 M NaCl-agar bridges. The Isc value is expressed as positive when the current flowed from the mucosa to serosa. Gt was measured by recording the current resulting from short-duration, square, bipolar voltage pulses (±5 mV) imposed across the tissue and then was calculated according to Ohm's law.

### ^22^Na^+^ Flux Measurements Across Colon and Ileum

The unidirectional transmural flux of ^22^Na^+^ was measured under short-circuit conditions. The mucosal-to-serosal (Jms) flux values were measured. Thirty minutes were allowed for the isotopic steady state to be reached after the mucosal side of the bathing solution was labeled with ^22^Na^+^. Ten samples (0.5 ml each) were taken from the unlabeled side at 10-min intervals and replaced with an equal volume of the unlabeled solution. The medium samples were assayed for ^22^Na^+^ by the liquid scintillation procedure. It is known that there are three Na^+^ absorption mechanisms in the mammalian distal colon [42]. The transporters responsible include: ENaC (inhibited by 10 µM benzamil), NHE2 (inhibited by 100 µM amiloride) and NHE3 (inhibited by 500 µM dimethylamiloride). To distinguish between the three possible mechanisms, we added the three inhibitors successively to the mucosal side and measured unidirectional ^22^Na^+^ flux. At first we added benzamil at time zero and then we added amiloride at time 60 minutes and finally at 90 minutes we added dimethylamiloride.

### Cells and Constructs

Madin-Darby Canine Kidney (MDCK) and opossum kidney (OK) cells were obtained from ATCC. The MDCK cells were stably transfected with NHE3 containing three tandem copies of the influenza virus HA epitope (YPYDVPDYA) inserted between the first and second membrane-spanning domains, between R38 and F39 (NHE3’_38HA3_), generated as described [43]. To select a stable line (MDCK-NHE3’_38HA3_), cells were cloned by limiting dilution in the presence of 500 µg/mL G418 and screened by immunofluorescence for expression of HA-tagged NHE3. MDCK and OK cells were maintained in a 1∶1 mixture of DMEM:F12 with 5% FBS in a 5% CO_2_ atmosphere. Experiments were performed at least 72 h after monolayers had reached confluence.

### SiRNA

Pooled siRNA targeting ezrin sequences GCUCAAAGAUAAUGCUAUGUU, GGCAACAGCUGGAAACAGAUU, CAAGAAGGCACCUGACUUUUU and GAUCAGGUGGUAAAGACUAUU and a control, non-targeting siRNA, (UAAGGCUAUGAAGAGAUACUU) were purchased from Dharmacon Inc. Cells were split at a high confluence, then 4 hours later, following the manufactures instructions, siRNA was transfected with Oligofectamine (Invitrogen). Cells were used for experimental purposes at 72–96 hours thereafter.

### Electron Microscopy

For scanning electron microscopy cells grown to confluence on transwell inserts were fixed at 4°C with 2% gluteraldyhyde for 30 min. They were post fixed with 1% osmium tetroxide followed by dehydration through a graded series of ethanol. Cells were then critical point dried, mounted on aluminum stubs and sputter coated with gold. The cells were viewed using an XL30 environmental SEM (ESEM) from FEI (Hillsboro, Oregon). Images were acquired with the secondary electron detector. For transmission electron microscopy, cells were fixed for 4 hours with 2% gluteraldyhyde, then postfixed in 1% OsO_4_ and 1.25% potassium ferrocyanide in sodium cacodylate buffer at room temperature for 2 h, stained en bloc for 1 h with 1% uranyl acetate in H_2_O, and then dehydrated and embedded in Epon resin (EMbed-812; Electron Microscopy Sciences). Sections (thickness, 70 to 80 nm) were collected on copper grids and stained with uranyl acetate and lead citrate. Sections were viewed using an FEI Tecnai 20 transmission electron microscope, and images were captured using a Gatan Dualview digital camera.

### Measurement of Na^+^/H^+^ Exchange Activity

NHE3 activity was assessed in MDCK-NHE3’_38HA3_ cells as the rate of Na^+^-induced pH_i_ recovery after an acid load. Dual excitation ratio determinations of the fluorescence of BCECF were used to measure pH_i_, as previously detailed [44]. Briefly, cells were grown to confluence on 25 mm glass cover slips, placed into Attofluor cell chambers and mounted on the stage of the microscope. Next, they were loaded with 5 µg/mL BCECF acetoxymethyl ester and pre-pulsed with 50 mM NH_4_Cl in HEPES-buffered RPMI at 37°C for 10 min for subsequent acid loading. Extracellular dye and NH_4_Cl were then washed away with Na^+^-free solution and Na^+^/H^+^ exchange was initiated by reintroduction of Na^+^ containing solution. Measurements of NHE3 activity were performed in the presence of 5 µM EIPA to obviate the contribution by NHE1. Intracellular pH (pH_i_) was calibrated by equilibrating the cells with K^+^-rich media titrated to defined pH values and containing 10 µg/mL nigericin [45]. Calibration was performed immediately after the recovery of pH_i_ for each experimental condition tested.

### Fluorescence Recovery After Photobleaching (FRAP)

Experiments were performed essentially as described previously [9]. In brief, MDCK-NHE3’_38HA3_ cells were transfected with either non-targeting control siRNA or siRNA targeting ezrin. 72 hrs later they were labeled with mouse anti-HA F(ab) fragment, (1∶300 dilution in RPMI) and then with secondary Alexa 488-conjugated goat anti-mouse F(ab) fragment (1∶500 dilution). Samples placed in Attofluor chambers were mounted on the stage of a confocal laser microscope (Zeiss LSM 510). The apical plane was brought into focus and two equal areas (2 µm diameter) defined. After acquiring two baseline fluorescence measurements, one of the selected areas was irreversibly photobleached and then the fluorescence of both areas measured over time. The fractional fluorescence recovery of the bleached area was determined relative to the average of the two pre-bleach measurements. The unbleached area was used to estimate possible bleaching incurred during image acquisition. Quantification of the maximal recovery rate was determined as in [46].

### NHE3 Quantification

Determination of surface-exposed and total cellular NHE3 was performed by an immunoperoxidase method, as detailed in [47]. Briefly, to quantify the amount of surface NHE3, cells were fixed with 4% paraformaldehyde on ice, blocked with 5% donkey serum for 30 min, and incubated with anti-hemagglutinin (HA) epitope antibody (1∶1,000 dilution) for 1 hr at room temperature. After washing the cells with PBS, they were incubated with a horseradish peroxidase-conjugated donkey anti-mouse antibody (1∶1,000 dilution) for 1 hr at room temperature. A similar protocol was used, in parallel, to quantify total NHE3, except that the cells were permeabilized with 0.1% Triton X-100 after fixation. After washing the excess secondary antibodies, the cells were incubated with 1 mL of OPD reagent for 10 min at room temperature. The reaction was stopped by adding 250 µL of 3 M HCl. The supernatant was collected, and absorbance was measured at 492 nm (*A*492) using a U-2000 spectrophotometer (Hitachi, Tokyo). In the range studied, *A*492 varied linearly with the amount of peroxidase bound. Background *A*492 was determined in parallel for every experiment by omission of the primary antibodies and was subtracted from each experimental determination.

### Other Methods

Immunostaining of surface, total and phosphorylated NHE3 was performed essentially as described in [9], [10], [39], [48]. Cells were visualized using a spinning disk inverted fluorescence microscope (Quorum) and image acquisition, quantitation and deconvolution were performed using Volocity™ V3.6 (Improvision) software. Extraction with Triton X-100 on whole cells and immunoblotting were performed as described previously [7]. An equal amount of protein was loaded per lane and all blots were stained with Ponceau Red before probing to ensure equal loading. Quantification of immunoblots was performed using Image-Pro Plus 4.1 software (MediaCybernetics, Silver Spring, MD, USA). Data are presented as means ± SEM of the number of determinations specified.

Details of the Material and Methods for Figures S1, S2, S4, S4 are provided in File S1.

### Data and Statistical Analysis

Data are presented as means ± SE. Comparison between two groups was made with Student’s *t*-test. Multiple comparisons were made by one-way analysis of variance (ANOVA) followed by Turky’s test unless stated otherwise. A P value of <0.05 was considered statistically significant.

## Results

### Ezrin Knockdown does not Alter NHE3 Activity in the Colon

The ezrin knockout mouse (*Ez^−/−^*) displays poorly developed intestinal microvilli that are shorter and thicker than their wild-type littermates [38]. NHE3 localization remained apical in these knockout animals. However, functional analysis of NHE3 activity was not performed and the animals fail to develop past weaning, making them difficult to study [40]. Consequently, an ezrin knockdown mouse (*Vil2^kd/kd^* ) was generated that despite severe growth retardation and mortality prior to weaning, showed a modest rate of survival (7%), eventually attaining approximately 75% of the body weight of their wild-type littermates by 7 weeks of age and survivng into adulthood. In these animals, ezrin protein expression was undectectable in all organs examined with the exception of the stomach which expressed <5% of the wild-type levels. Despite their normal appearance, these animals demonstrate severe achlorhydria due to a defect in the formation of the apical canaliculi of gastric epithelial cells [40]. However, analysis of NHE3 localization and function has not been performed.

To evaluate the impact of ezrin knockdown on NHE3 activity *in vivo,* we first measured ^22^Na^+^ flux across the ileum of Vil2^kd/kd^ mice (Figure S1 and Table S1). Under baseline conditions there was decreased Na^+^ flux in the *Vil2* ^kd/kd^ mice compared to wild-type animals. The dimethylamiloride sensitive ^22^Na^+^ flux was also lower in *Vil2* ^kd/kd^ mice, consistent with reduced NHE3 activity due to either a direct or indirect effect of the absence of ezrin. We also observed reduced conductance across ileal preparations from *Vil2* ^kd/kd^ mice compared to wild-type animals, 20.6±1.4 vs 30.1±5.3 mS/cm^2^ respectively.

**Figure 1:**
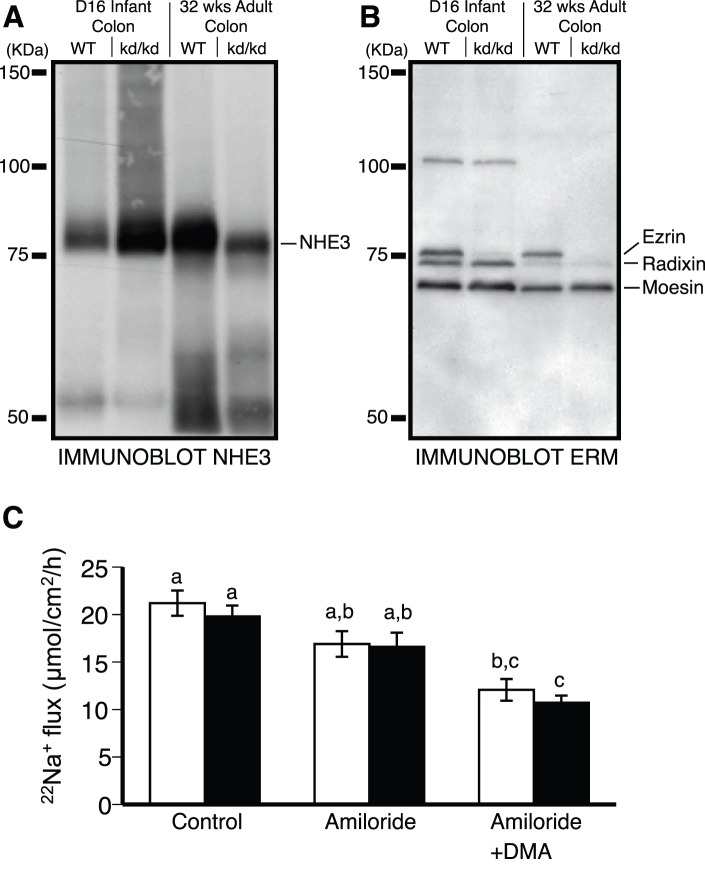
Ezrin knockdown does not alter NHE3 activity *in vivo*. Immunoblot analysis of colon from wildtype and *Vil2^kd/kd^* mice at day 16 (D16) pups and at 32 weeks of age (32 wks) adults for A) NHE3 and B) total ERM. C) Assessment of Na^+^ flux across colonic mucosa of either wild-type (WT) (white bars, n = 6) or *Vil2^kd/kd^* (black bars, n = 5) mice in the presence of 10 µM benzamil (ENaC inhibited, Control) and then also in the presence of 100 µM amiloride (NHE2 inhibited, Amiloride) and finally in the presence of the previous two drugs as well as 500 µM dimethylamiloride (NHE3 inhibited, Amiloride+DMA). Columns not sharing the same lower-case are significantly different by ANOVA.

We also examined the activity of NHE3 in colon of *Vil2^kd/kd^* animals. To our surprise, the activity of NHE3 was unaltered between the wild-type and *Vil2^kd/kd^* animals (Figure 1C; dimethylamiloride sensitive ^22^Na^+^ flux, 4.9±1.0 and 5.9±1.0 µmol/cm^2^/h, wild-type and *Vil2^kd/kd^* respectively, P = 0.59 ), despite a decrease in the amount of NHE3 expressed at 32 weeks of age (Figure 1A). Basal electrical parameters were not significantly different between the wild-type and *Vil2^kd/kd^* animals (Table 1). Increased ENaC activity has been observed in the colon of NHE3 knockout animals (5). To elimate the possibility that increased ENaC activity was somehow masking altered NHE3 activity, benzamil (10 µM) was added to the luminal compartment throughout our ^22^Na^+^ flux studies inorder to inhibit ENaC activity. The presence of benzamil did not alter Na^+^ flux across the colon of *Vil2^kd/kd^* mice relative to wild-type animals (Figure 1C, Control). Ultimately the interpretation of Na^+^ flux studies in *Vil2^kd/kd^* mice is complicated by the expression of significant amounts of radixin and moesin in the colon of these animals (Figure 1B), as there may be functional redundancy between ezrin and these other ERMs. Consistent with this possibility, we were able to co-immunoprecipitate NHE3 with all three ERMs (Figure S2).

**Table 1:** Comparison of basal electrical parameters and benzamil sensitive Isc between wild-type and ezrin knock-down mouse colon.

	Wild-type	*Vil2^kd/kd^*	
**Basal Isc** (µA/cm^2^)	33.2±2.2 (6)	44.9±19.1 (5)	P = 0.55
**Basal Gt**(mS/cm^2^)	14.1±2.2 (6)	12.5±1.6 (5)	P = 0.53
**Benzamil sensitive Isc**(µA/cm^2^)	6.0±6.1 (6)	8.3±4.6 (5)	P = 0.75

Each value represents the mean ± S.E.M. Isc = short circuit current. The number of preparations are indicated in parentheses. Three mice were used for each group.

### Ezrin Knockdown in a Cell Culture Model Lacking Radixin and Moesin

**Figure 2:**
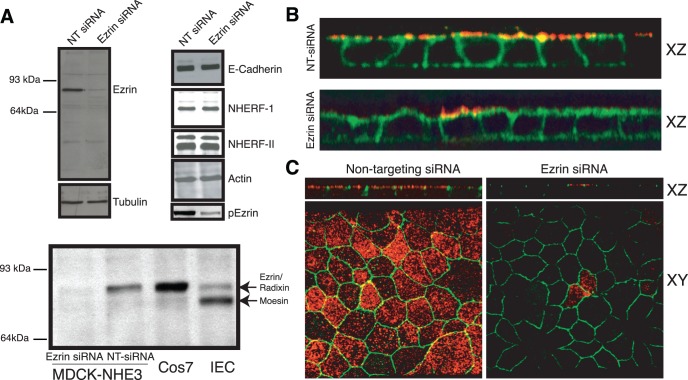
Ezrin knockdown *in vitro* does not induce radixin or moesin expression, nor alter epithelial morphology. A) Representative immunoblots (n≥3) for ezrin, tubulin, actin, E-cadherin, NHERF-I, NHERF-II and phosho-ezrin of whole cell lysates (NHE3’_38HA3_,cells) after treatment with non-targeting siRNA or siRNA targeted against ezrin. Whole cell lysate from Cos7 and IEC cells were included as controls. B) XZ reconstruction of confocal stacks of a monolayer of NHE3’_38HA3_ cells labeled with ezrin (red) and treated with Alexa488-conjugated phalloidin (green) after treatment with non-targeting siRNA or siRNA targeted against ezrin. C) XZ reconstruction of confocal stacks, and apical XY images, of a monolayer of NHE3’_38HA3_ cells labeled with ezrin (red) and ZO-1 (green) after treatment with non-targeting siRNA or siRNA targeted against ezrin.

To eliminate the confounding presence of other ERM proteins, we sought an epithelial cell culture model largely devoid of both radixin and moesin. This would enable the independent assessment of the contribution of ezrin to NHE3 apical localization, retention and activity. For this purpose we employed the MDCK-II cell line, as it has been reported to have very low levels of both radixin and moesin, whilst retaining significant expression of ezrin [49]. This was confirmed experimentally using a pan-ERM antibody (Figure 2A). As MDCK cells do not express NHE3 we used a cell line engineered to stably express NHE3 with an exofacial tri-hemagluttin tag [9]. The presence of this tag has the added benefit of facilitating the distinction between NHE3 within the apical plasma membrane from NHE3 in subapical endomembranes.

The expression of ezrin in this cell line was suppressed using small interference RNAs. As shown in Figure 2A, the level of ezrin was decreased to <10% of the non-targeting siRNA transfected level, without inducing the expression of either radixin or moesin. Knockdown of phospho-ezrin was slightly less effective, to ≈ 20% the non-targeting transfected cells (Figure 2A). It should be noted that immunoblot analysis measures the level of expression of ezrin within the entire population of cells. To asses ezrin expression in single cells, immunofluorescent microcopy was performed after siRNA treatment. These experiments revealed that in >90% of the cells, ezrin expression is completely eliminated (Figures 2B and C), whilst the remaining 10% of cells had a normal level of ezrin. Next, the specificity of the siRNA for ezrin was assessed. Immunoblot analysis of actin, tubulin, E-cadherin, ZO-1, NHERF-I and NHERF-II revealed comparable levels of expression between non-targeting and ezrin targeting siRNA transfected cells. We confirmed and extended this observation by performing immunofluorescent microscopy on a monolayer of cells transfected with non-targeting siRNA or siRNA targeted against ezrin. This revealed that in the absence of ezrin, ZO-1 retained its localization to tight junctions, and the cells retained their columnar epithelial morphology (Figures 2B and C).

**Figure 3:**
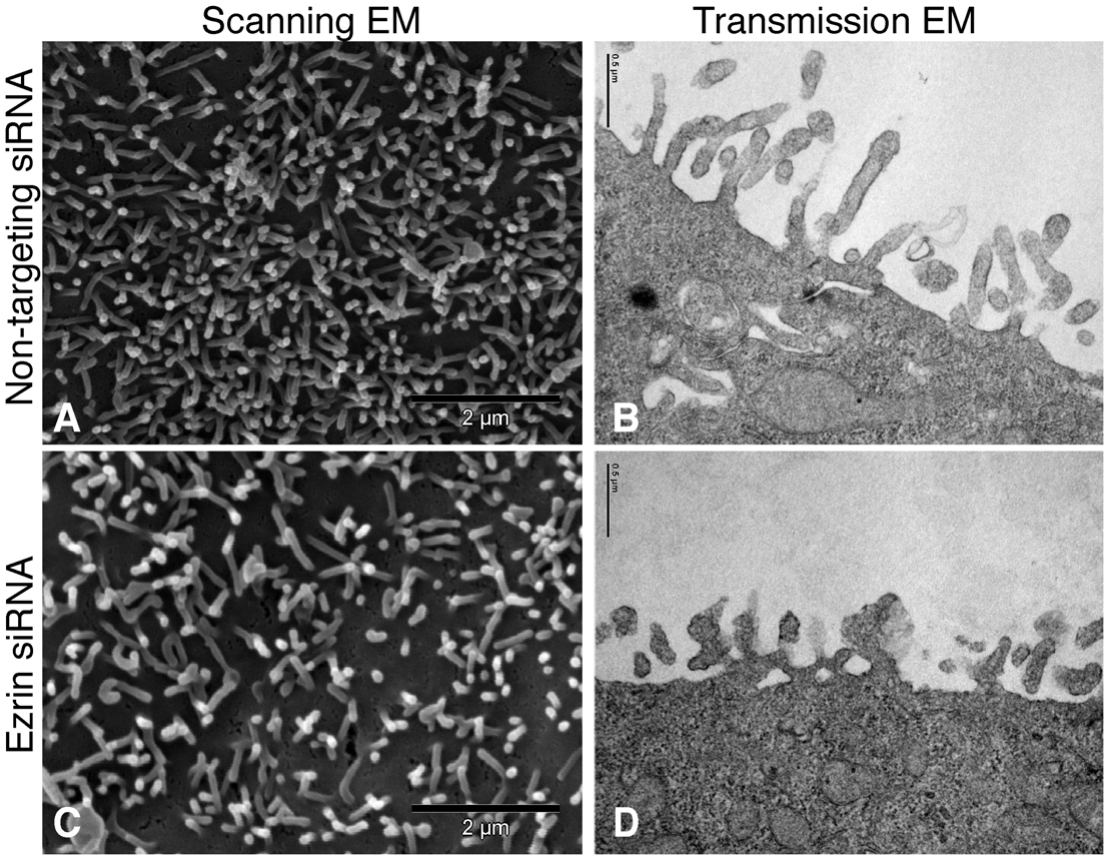
Ezrin knockdown causes shortened, thicker and fewer microvilli. Representative scanning (left two panels) and transmission (right two panels) electron micrographs of the apical surface of MDCK cells stably expressing NHE3’_38HA3_ after treatment with non-targeting siRNA (top two panels) or siRNA targeted against ezrin (bottom two panels).

Ultrastructural analysis of ezrin knockout *in vivo* revealed microvillar formation abnormalities that resulted in shortened and thicker microvilli [38]. We enquired whether these abnormalities were present in our *in vitro* cell culture model devoid of detectable ERM expression. Scanning and transmission electron microscopy was consequently completed on cells that had been transfected with non-targeting or siRNA targeted against ezrin (Figure 3). The microvilli in the ezrin siRNA-treated cultures appeared to be fewer in number as well as thicker and shorter (Figure 3C and D). When quantified by counting the number of microvilli on the cell surface of 20 representative scanning electron micrographs the number of microvilli on cells transfected with non-targeting siRNA was significantly greater than the cells transfected with siRNA against ezrin (395±16 *vs.* 329±18, p<0.01). Similarly, when the microvilli observed on transmission electron micrographs were measured and compared between cells transfected with non-targeting siRNA and cells transfected with siRNA targeted against ezrin, the latter population had shorter (237±13 vs. 157±8 nm, p<0.01) and thicker (39±2 vs. 55±6 nm, p<0.05) microvilli.

### Ezrin is not Necessary for the Apical Localization of NHE3

**Figure 4:**
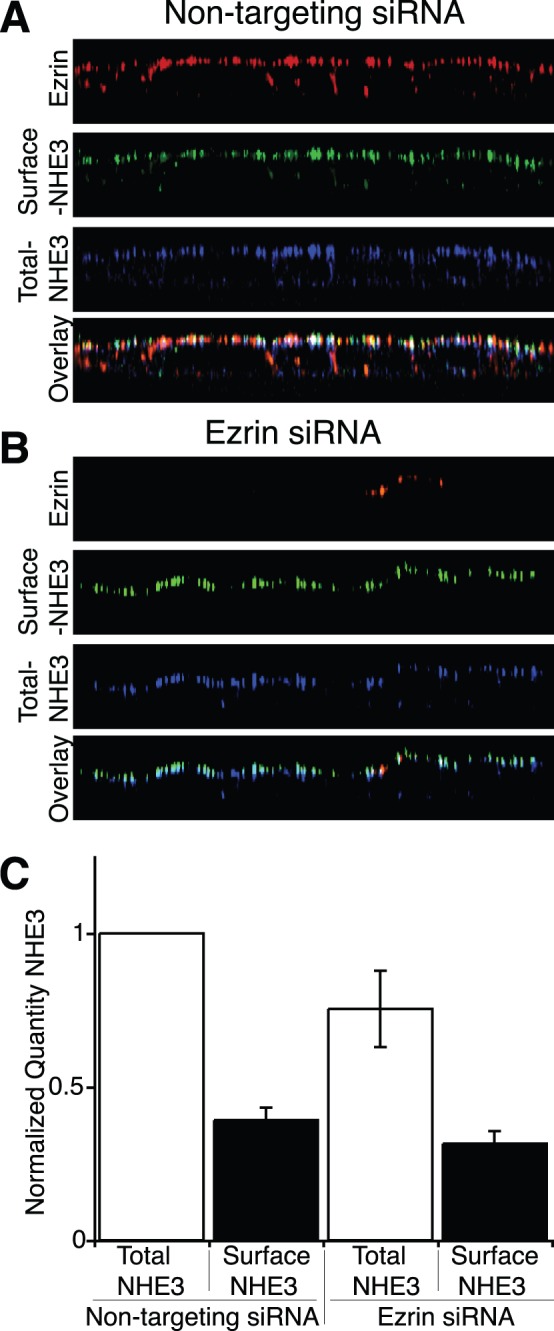
Ezrin is not necessary for the apical localization of NHE3. XZ reconstruction of confocal stacks of a monolayer of NHE3’_38HA3_ cells labeled with ezrin (red), surface NHE3 (green) and total NHE3 (blue) after treatment with non-targeting siRNA A) or siRNA targeted against ezrin B). C) Quantification by an immunoperoxidase based assay of surface and total NHE3 after treatment with non-targeting siRNA or siRNA targeted against ezrin (n = 4 per condition).

The subcellular localization of NHE3 in the absence of ezrin was next examined. The exofacial tri-hemagluttin tag was utilized to perform immunofluorescent microscopy on the surface and the endomembrane fraction of exchangers in the same cell. This was accomplished by first immunolabelling the cell surface population of intact cells with a green fluorophore. The cells were then permeabilized with a weak detergent and the entire population of exchangers labeled with a different colored fluorophore (far red, psuedocolored blue) whilst simultaneously immunolabeling ezrin (red). We did this on cells transfected with non-targeting siRNA and siRNA targeted against ezrin. Despite the complete absence of ezrin in >90% of the cells transfected with siRNA against ezrin the distribution of NHE3 appeared unaltered from cells transfected with non-targeting siRNA (Figure 4A and B). The fraction of exchangers in the apical membrane, as a fraction of the total number of exchangers, was quantified using an independent spectrophotometric assay (Figure 4C). These measurements revealed a slight decrease in the total amount of NHE3, yet no discernable difference was detected in the fraction of NHE3 at the apical plasma membrane in the absence of ezrin, consistent with the animal data (Figure 1A and [38]).

### Ezrin is not Required for the Association of NHE3 with the Apical Actin Cytoskeleton

**Figure 5:**
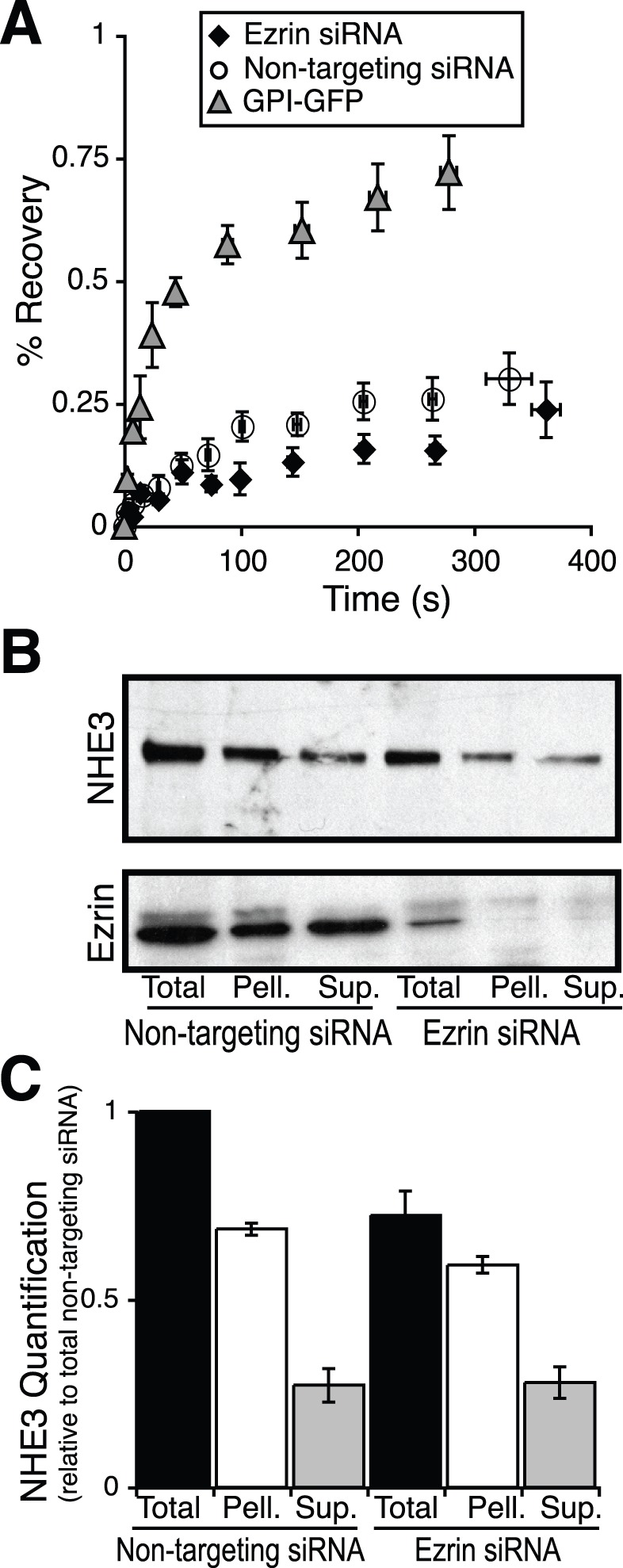
Ezrin does not form a functional link between NHE3 and the actin cytoskeleton. A) Fluorescence recovery after photobleaching (FRAP) analysis of GPI linked GFP (grey triangles) and NHE3 after treatment with non-targeting siRNA (open circles) or siRNA targeted against ezrin (black diamonds), n≥20 cells per condition. B) Representative immunoblots and C) their quantification (n = 3) of triton-X 100 soluble (Sup.) triton insoluble (Pell.) and total NHE3 after treatment with non-targeting siRNA or siRNA targeted against ezrin.

One model of NHE3 apical localization proposes that it is tethered to the actin cytoskeleton via a link to NHERF-I and then ezrin. Interestingly, another study suggested that ezrin may bind directly to NHE3, thereby tethering the transporter to the actin cytoskeleton [31]. Both models predict that ezrin serves as an anchor between NHE3 and the actin cytoskeleton. This prediction implies that ezrin prevents both the lateral mobility of NHE3 in the plane of the apical membrane and the solubility of NHE3 in the weak detergent Triton X-100. To assess the first prediction we measured the mobility of NHE3 in the plane of the apical plasma membrane using the technique of fluorescent recovery after photobleaching (FRAP). We were able to specifically label the apical plasma membrane population of NHE3 using the exofacial tag in cells transfected with either non-targeting siRNA or siRNA targeted against ezrin. Under control conditions NHE3 is largely immobile in the plane of the plasma membrane (Figure 5A and [9], [22]). Unexpectedly NHE3 remained immobile in the plane of the apical plasma membrane when ezrin was knocked down (Maximum recovery 37±5% in the non-targeting transfected cells *vs* 26±5% in the cells transfected with siRNA targeted against ezrin), suggesting that ezrin is not responsible for the functional association between NHE3 and the actin cytoskeleton. This immobility is not a consequence of our experimental system, as GPI-GFP is largely mobile in the apical membrane when expressed in MDCK-II cells, maximum recovery to 74±4% to the prebleach state (Figure 5A).

To test the second prediction we performed differential solubility experiments on cells transfected with non-targeting and siRNA targeted against ezrin. Under baseline conditions NHE3 is largely Triton X-100 insoluble (Figure 5B and [25]), as it is tethered to the actin cytoskeleton. Consistent with the FRAP data, the fraction of exchangers soluble in Triton X-100 was unaltered when ezrin was depleted to less than 20% the non-targeting transfected population (Figure 5B and C). Together these findings demonstrate that ezrin is not responsible for the tethering of NHE3 to the actin cytoskeleton.

### Ezrin Knockdown does not Alter Baseline NHE3 Activity

**Figure 6:**
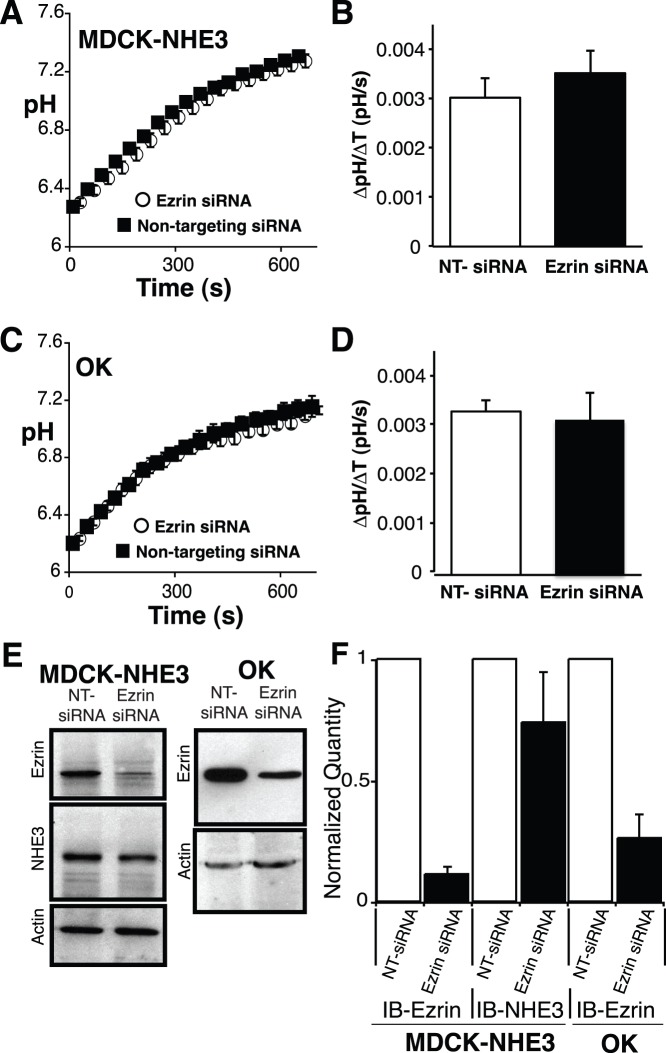
Ezrin is not required for NHE3 activity. The activity of NHE3 was measured as the sodium dependent recovery of pH after ammonium prepulse in A) NHE3’_38HA3_, cells and C) OK cells after transfection with either non-targeting siRNA (black squares) or siRNA targeted against ezrin (open circles) and found to be identical. Quantification of sodium dependent recovery of pH for the first 60 s of recovery after switching to sodium containing medium for NHE3’_38HA3_ cells B) and OK cells D). The rate was normalized to cell surface fraction of NHE3 for NHE3’_38HA3_ cells. After each measurement all cells on the coverslip were solubilized in Laemmli buffer and immunoblot analysis was performed for ezrin and actin (OK cells) and also NHE3 (NHE3’_38HA3_ cells). E) Representative immunoblots and F) quantification of all immunoblots. The data are represented as the mean ± SEM of at least 12 different experiments per condition.

Although the fraction of exchangers in the apical plasma membrane is unaltered (Figure 4A–C), this provides no insight into the activity of the exchangers residing in the apical membrane. The binding of ezrin to NHE3 has been suggested to alter the activity of the exchanger [31]. Thus, despite normal localization, the baseline activity of NHE3 may be altered. To assess this possibility we performed ezrin knockdown and measured the activity of NHE3 as sodium dependent recovery of pH following intracellular acidification imposed by prepulsing the cells with ammonium chloride. As a control we performed similar experiments in cells transfected with non-targeting siRNA. As MDCK cells express NHE1 endogenously; all functional measurements were made in the presence of 5 µM EIPA, a concentration that inhibits NHE1 and NHE2, but not NHE3 [50], [51]. Contrary to the previous report [31], we found no difference in NHE3 activity between cells transfected with non-targeting or siRNA targeted against ezrin (Figure 6A and B). These findings are consistent with the results obtained from measurements made on whole colon from the ezrin knockdown mouse (Figure 1C). To exclude the possibility that ezrin knockdown may alter the cellular buffering capacity, we measured it in cells transfected with non-targeting or siRNA targeted against ezrin. There was no difference, 18±3 *vs* 19±4 mM/pH unit respectively.

A limitation of this experimental design is the inability to detect which cells had ezrin knocked down at the time of NHE3 activity measurement. We therefore measured the activity of NHE3 in whole fields of cells (approximately 25 cells/field) multiple times (>12 fields/condition) and averaged the activity of NHE3 from this large number of cells (>300 cells/condition). Further, to insure that ezrin had indeed been knocked down in the population we had measured activity on, we quantified ezrin expression by immunoblot after each measurement (Figure 6E and F). Finally, to confirm that these results were not due to the expression of exogenous NHE3 in an engineered cell culture model, we repeated the measurements in a cell line that expresses NHE3 endogenously, OK cells. Consistent with both the animal (Figure 1C), and the other cell culture model data (Figure 6A and B), there was no detectable difference between the rate of pH recovery mediated by NHE3 in the presence of normal or greatly reduced expression of ezrin in OK cells (Figure 6C–F).

### Ezrin is Necessary for the cAMP Mediated Inhibition of NHE3, but not for PKA Mediated Phosphorylation of the Exchanger

**Figure 7:**
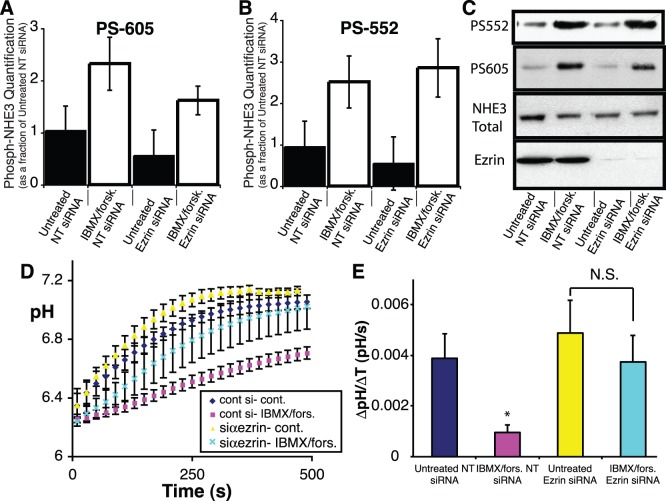
Ezrin is necessary for cAMP mediated inhibition of NHE3, but not for the phosphorylation of NHE3 at serines 552 or 605. C) Representative immunoblots and quantification (n = 3) of the phosphorylation of NHE3 at serine 605 A) and 552 B) of cells treated with forskolin/IBMX or vehicle, after transfection with either non-targeting siRNA or siRNA targeted against ezrin. D) NHE3 activity after treatment with forskolin/IBMX (pink squares and light blue crosses) or vehicle (yellow triangles or blue diamonds), after transfection with either non-targeting siRNA (blue diamond or pink square) or siRNA targeted against ezrin (yellow triangles or light blue crosses). E) Quantification of sodium dependent recovery of pH over the first 60 s of recovery after switching to sodium containing medium for the traces displayed in D. The data are represented as the mean ± SEM of at least 8 different experiments per condition, * represents a p value <0.05.

The association between the cytosolic c-terminus of NHE3 and ezrin has been proposed to mediate the localization of PKA to, and phosphorylation of, the cytosolic c-terminus of NHE3. Thus, ezrin is thought to be a PKA anchoring protein or AKAP [18]. The PKA mediated phosphorylation of NHE3 at serine residues 552 and 605 has been well characterized and is known to be associated with inhibition of NHE3 activity [14], [15]. To ascertain whether ezrin is necessary for the cAMP mediated phosphorylation and subsequent inhibition of NHE3 in our model system, cAMP levels were elevated by pretreating cells with forskolin to stimulate adenylate cyclase activity and IBMX to inhibit the breakdown of cAMP, and then NHE3 activity was assessed. This was performed in both model cell culture systems, MDCK-NHE3’_38HA3_ and OK cells. Both systems displayed an inhibition of NHE3 activity when pretreated with IBM/forskolin. We proceeded to perform the same experiments on cells that had been transfected with either non-targeting or siRNA directed against ezrin. Consistent with the proposed model in both OK cells (Figure 7D and E) and MDCK-NHE3’_38HA3_ cells (Figure S3) ezrin knockdown significantly attenuates cAMP mediated inhibition of NHE3.

The mechanism responsible for cAMP mediated inhibition of NHE3 was initially thought to be secondary to the direct phosphorylation of NHE3 at serines 552 and 605 by PKA [15]. However, subsequent studies have demonstrated that phosphorylation of NHE3 at serines 552 and 605 does not always directly correlate with changes in NHE3 activity [48]. To ascertain whether this mechanism accounted for the cAMP mediated inhibition of NHE3, the phosphorylation status of serines 552 and 605 was determined after treatment with IBMX/forskolin in the presence or absence of ezrin. This was accomplished using phosphospecific antibodies to phosphoserine 552 and 605 of NHE3 [39]. Surprisingly, despite the fact that ezrin knockdown largely prevented cAMP mediated inhibition of NHE3, depletion of ezrin failed to prevent the phosphorylation of NHE3 at serine 552 and 605 (Figure 7A–C). We confirmed this finding by performing immunofluorescent microscopy on cells treated with IBMX/forskolin after transfection with non-targeting or siRNA targeted against ezrin. These experiments confirmed that in the absence of ezrin, NHE3 can be phosphorylated at serine 552 (Figure S4.A). Finally to clarify whether ezrin was capable of altering the localization of PKA, thereby acting as an AKAP, we performed similar immunofluorescent microscopy analysis of the PKA-R_II_ subunit (the isoform known to bind ezrin [18], [52]). This revealed no alteration in the localization of PKA-R_II_, (Figure S4.B) despite ezrin depletion. These findings support our observation that, in the absence of ezrin, PKA is still able to localize to the apical plasma membrane of epithelia and, in the presence of cAMP, phosphorylate NHE3.

## Discussion

Using a variety of biochemical and immunofluorescent microscopic methodologies we demonstrate that ezrin is not necessary for the apical localization, tethering to the apical actin cytoskeleton, nor for baseline activity of NHE3 in cell culture. This is unlikely to be due to an increased expression of, or functional redundancy between ezrin and the other ERM proteins since the cell culture model employed lacked detectable levels of radixin and moesin, even in the absence of ezrin. Our findings, while contrary to current dogma, are consistent with the limited NHE3 localization studies performed on both the NHERF-1 [37] and the ezrin knockout mouse [38]. Whilst ezrin has been shown to bind the cytosolic C-terminus of NHE3 directly [31], this interaction is clearly not the only mechanism responsible for the retention of NHE3 at the apical plasma membrane nor does it appear to be the only molecule that mediates the interaction between NHE3 and the apical actin cytoskeleton.

What then is responsible for the localization and retention of NHE3 at the apical membrane? One possibility is another linker protein. Numerous linker proteins are found in renal and intestinal epithelium that may serve this function; however at present, there are no data to support a preference for any one candidate. We would be remiss not to consider other possible mechanisms of NHE3 apical localization and retention. An interaction between the membrane domain of NHE3 and another apicaly retained protein, which is in turn linked to the actin cytoskeleton, either directly or as part of a large protein complex, could explain the observed results. Alternatively NHE3 may not be directly tethered to the actin cytoskeleton at all, but could exist within an apical actin fence that restricts the mobility/solubility of NHE3 as has been described for the potassium channel Kv2.1 [53].

Our results, however, do support a role for ezrin in the regulation of NHE3 activity. Previous studies have shown a role for ezrin in Na^+^ glucose cotransport mediated activation of the exchanger [54] and in the activation of NHE3 via CHP-1 [55]. Specifically, we demonsrate that ezrin is necessary for cAMP mediated inhibition of NHE3. The mechanism of this inhibition has been proposed to be through a direct phosphorylation of NHE3 on the cytosolic serine residues 552 and 605 by PKA [14], [15]. Ezrin has been suggested to mediate the interaction between PKA and the cytosolic c-terminus of NHE3, enabling phosphorylation of its c-terminus. Our findings, using both biochemical and immunofluorescent microscopic techniques, demonstrate that ezrin is neither required for the apical localization of the regulatory subunit of PKA (RII, the subunit known to bind ezrin directly [18]) nor for the phosphorylation of NHE3 at the PKA consensus residues. This is consistent with previous data that clearly separates cAMP induced inhibition of NHE3 (which is ezrin dependent, at least in part) from cAMP induced phosphorylation of the c-terminus of NHE3 (which occurs in an ezrin independent fashion) [48]. Therefore ezrin does not act as an AKAP, at least in this context.

How then does cAMP inhibit NHE3 activity, if not through the direct phosphorylation of NHE3 on its cytosolic C-terminus? There exist at least two potential explanations. The first is that PKA phosphorylates another protein in a ezrin dependent fashion; this event in turn results in the inhibition of NHE3 activity. The other possible explanation is that increased levels of cAMP may inhibit NHE3 independently of PKA. Consistent with the latter possibility is the observation that the exchange protein directly activated by cAMP, termed EPAC, can inhibit NHE3 activity independently of PKA [56]. Finally, it should be noted that whilst phosphorylation of NHE3 at serine residues 552 or 605 is not sufficient for inhibition of NHE3 activity, we have provided no evidence to suggest that this event is not necessary. Indeed, several reports from the literature have shown that these phosphorylation events are in fact necessary for cAMP mediated inhibition of NHE3 activity [14], [15].

The animal and cell culture data (Figures 1, 5 and 6) suggest a slight decrease in the quantity of NHE3, despite normal localization and function. How can we reconcile fewer exchangers at the plasma membrane despite identical NHE3 activity? Our regulation data offer some insights. Phosphorylation of NHE3 is known to be associated with inhibition of NHE3. We have shown that under baseline conditions there is a slight decrease in the phosphorylation of NHE3 at serines 552 and 605. We suggest therefore that, although in the absence of ezrin there are a decreased total number of exchangers at the plasma membrane, the number of functional exchangers (*i.e.* not phosphorylated) is in fact similar. This would account for our observed results, although any mechanism altering the activity of individual exchangers in the membrane that is perturbed by the absence of ezrin is equally plausible. What accounts for the decreased NHE3 expression in the absence of ezrin? This could be explained by a decrease in the number of microvilli. We have shown previously that NHE3 is preferentially associated with microvilli [9]. Here we demonstrate a reduced number and size of microvilli consistent with published results in the ezrin knock out mice [38]. However, ezrin knockdown mice did not appear to have fewer microvilli in their brush border [40]. Might a lack of the preferred domain where NHE3 is retained result in its redistribution to the endomembrane compartment where an excess of exchanger is subsequently degraded? Evidence to support this hypothesis is currently lacking.

It has been reported that the knockdown of all three ERM proteins prevents the formation of microvilli in thymoma cells (L5178Y) [57]. We report here epithelial cells that lack detectable expression of ezrin, radixin and moesin yet retain a limited number of shortened, wider microvilli. These results are readily explained by looking at the duration of ERM knockdown in the different cells. Our cells showed a decreased number, shorter and wider microvilli after 4 days of ezrin depletion. This is in fact the same phenotype observed in the previous report after 4 days of knockdown [57]. It was not until after 6 days of ERM depletion that cells lacking microvilli were detectable. In our model, ezrin expression begins to recover by day 5 after transfection. As MDCK cells grow in confluent monolayers, we were unable to efficiently transfect siRNA into our model system a second time to maintain knockdown for >4 days. Taken together, these findings confirm a lack of ERM expression in our system and provide insight into the stability of microvillar structure.

Our *in vivo* data argue for and agaist the involvement of ezrin in regulating baseline NHE3 activity. The ileal studies are consistent with reduced baseline NHE3 activity while the colonic studies failed to support this. Why this is the case is not clear. The small number of animals employed for each study may be contributing to the large variance observed, which in turn may be masking a significant difference. Although it did not reach statistical significance, basal Isc was increased in the ileum and colon of *Vil2* ^kd/kd^ mice. This finding is consistent with increased Cl^−^ secretion in the *Vil*2^kd/kd^ mice, that may either directly or indirectly affect basal NHE3 activity.

In summary our data suggest that ezrin is not the only functional linker between NHE3 and the actin cytoskeleton and consequently not the only determinant of the apical localization of NHE3. We also provide evidence that ezrin is not acting as an AKAP at the apical plasma membrane, although ezrin is involved in the regulation of NHE3 activity. Our findings also question the role of the NHERFs in NHE3 apical localization, as linkage through ezrin to the actin cytoskeleton has been repeatedly proposed to mediate the apical localization of NHE3 [19], [20], [21]. These findings have physiological significance, as altered NHE3 activity has been implicated in the pathogenesis of excess salt and water retention associated with some forms of hypertension [58], [59]. An understanding of the mechanisms responsible for the apical localization of NHE3 is prerequisite to understanding its regulation and consequently its contribution to volume overload and hypertension. Consideration of this new information may better enable the manipulation of these processes therapeutically to treat such diseases as idiopathic hypertension.

## Supporting Information

Figure S1 **Assessment of Na^+^ flux across ileal mucosa of either wild type (WT) (white bars, n = 5 preparations, from 3 mice) or** ***Vil2^kd/kd^*** **(black bars, n = 6 preparations from 3 animals) mice in the presence of 10 µM benzamil (ENaC inhibited, Control) and then also in the presence of 100 µM amiloride (NHE2 inhibited, Amiloride) and finally in the presence of the previous two drugs as well as 500 µM dimethylamiloride (NHE3 inhibited, Amiloride+DMA).** * Represents p<0.05 compared to wild type control and # represents p<0.05 compared to kd control by 2 way ANOVA.
(EPS)

Figure S2 **NHE3 binds to all three ERMs A) MDCK cells stably expressing NHE3’_38HA3_ (MDCK-NHE3’_38HA3_) were transiently transfected with either: ezrin, radixin or moesin (all containing a c-terminal myc tag).** After cell lysis, the proteins were either directly resolved on SDS-PAGE (input) or immunoprecipitated (IP) with a rat anti-HA antibody before SDS-PAGE. After proteins were transferred to nitrocellulose membranes, samples were blotted with rabbit anti-myc antibody followed by anti-rabbit HRP antibody. B) Alternatively, cell lysate obtained as per A was either directly resolved on SDS-PAGE (input) or immunoprecipitated (IP) with the rabbit anti-myc antibody before SDS-PAGE. After transfer, the samples were blotted with mouse anti-HA antibody followed by anti-mouse HRP antibody. Co-immunoprecipitations performed in the absence of primary antibody (No 1°) are included as a control. Displayed are representative blots from three separate experiments.
(EPS)

Figure S3 A) NHE3 activity measured in MDCK-NHE3’_38HA3_ cells after treatment with forskolin/IBMX (red squares and purple Xs) or vehicle (blue diamonds or green triangles), after transfection with either non-targeting siRNA (blue diamonds or red squares) or siRNA targeted against ezrin (green triangles or purple Xs). B) Quantification of sodium dependent recovery of pH over the first 60 s of recovery after switching to sodium containing medium for the traces displayed in A (n is >6 per condition, * represents p<0.05).
(EPS)

Figure S4 A) XZ reconstruction of confocal stacks of a monolayer of NHE3’38HA3 cells labeled with ezrin (green) and phospho-serine 552 of NHE3 (red) after treatment with non-targeting siRNA or siRNA targeted against ezrin. B) XZ reconstruction of confocal stacks of a monolayer of NHE3’38HA3 cells labeled with ezrin (red) and the regulatory subunit of PKA (RII) (green) after treatment with non-targeting siRNA or siRNA targeted against ezrin.
(EPS)

Table S1 **Comparison of basal electrical parameters and benzamil sensitive Isc between wild-type and ezrin knock-down mouse ileum.**
(DOCX)

File S1 **Supplemental materials and methods.**
(DOCX)
