# Supplementary material for: Ezrin Is Required for the Functional Regulation of the Epithelial Sodium Proton Exchanger, NHE3
Source: PLoS One. 2013 Feb 6;8(2):e55623. doi: 10.1371/journal.pone.0055623 (PMC3566197; doi:10.1371/journal.pone.0055623)
Supplement: Table S1 — Comparison of basal electrical parameters and benzamil sensitive Isc between wild-type and ezrin knock-down mouse ileum. (DOCX) [file pone.0055623.s005.docx]

**Table S1.** Comparison of basal electrical parameters and benzamil sensitive Isc between wild-type and ezrin knock-down mouse ileum

|  | **Wild-type** | ***Vil2^kd/kd^*** |  |
| --- | --- | --- | --- |
| **Basal Isc** (μA/cm^2^) | 17.7 ± 4.1 (5) | 31.1 ± 4.4 (6) | P = 0.19 |
| **Basal Gt** (mS/cm^2^) | 30.1 ± 5.3 (5) | 20.6 ± 1.4 (6) | P = 0.06 |
| **Benzamil sensitive Isc** (μA/cm^2^) | Not detected (5) | Not detected (6) |  |

Each value represents the mean ± S.E. Isc = short circuit current. The number of preparations is indicated in parentheses. Three mice were used for each group.
