# Supplementary material for: Ezrin Is Required for the Functional Regulation of the Epithelial Sodium Proton Exchanger, NHE3
Source: PLoS One. 2013 Feb 6;8(2):e55623. doi: 10.1371/journal.pone.0055623 (PMC3566197; doi:10.1371/journal.pone.0055623)
Supplement: File S1 — Supplemental materials and methods. (DOCX) [file pone.0055623.s006.docx]

**Text File S1**

**Supplemental Materials and Methods**

*Immunoprecipitation.* MDCK cells stably expressing NHE3’_38HA3_ (MDCK-NHE3’_38HA3_) were transfected by electroporation (Neon Transfection system, Invitrogen) with either: ezrin, radixin or moesin (all were mouse, c-terminally myc tagged and were from Origene Technologies Inc.). After 24hrs the cells were lysed in PBS containing 1% Triton X-100 and protease inhibitors (1 μg/ml aprotinin, 2 μg/ml leupeptin, 1 μg/ml pepstatin A and 100 μg/ml PMSF). Aliquots of cell lysate were saved as total fraction, the remaining cell lysates were incubated with rabbit anti-myc antibody (Covance, Princeton, NJ), (or no antibody as a negative control) followed by precipitation with protein G-Sepharose (Thermo Scientific, Rockford, IL). The bound proteins were eluted with Laemmli buffer and then immunoblotted and detected with a mouse anti-HA antibody (Covance, Princeton, NJ) followed by an anti-mouse antibody coupled to horseradish peroxidase (HRP). Alternatively, proteins in cell lysates were immunoprecipitated with a rat anti-HA antibody (Roche Diagnostics, Basel, Switzerland) and detected using the rabbit anti-myc antibody.

*Immunofluorescence microscopy*. Cells grown on 6.5 mm, 0.4 μm pore diameter, transwells (Costar), 72 hrs after treatment with siRNA were fixed with 2% paraformaldehyde on ice for 30 min. They were then permeabalized with 0.1% Triton X-100 in PBS with 5% milk for 1 hr at room temperature. Primary antibodies were added at a concentration of 1:1000 monoclonal anti-ezrin (Covance) and 1:250 anti-PKA RII (BD Transduction Laboratories Inc.) for 1 hr at room temperature. Polyclonal anti-ezrin (1:500) and anti-PS552 NHE3 (1:100) were incubated for 4 hrs at room temperature. Secondary antibodies, donkey anti-rabbit Cy2 and donkey ant-mouse Cy3 (both from Jackson Immuno Research Inc.) were added at a concentration of 1:1000 at room temperature for an hour. Finally, filters were mounted on glass slides and imaged with a spinning disk inverted fluorescence microscope (Quorum).
